# THE ASSOCIATION OF PHYSICAL ACTIVITY AND MORTALITY AMONG RECENTLY HOSPITALIZED OLDER ADULTS WITH DEMENTIA

**DOI:** 10.1093/geroni/igae098.3494

**Published:** 2024-12-31

**Authors:** Brittany Burch, Barbara Resnick

**Affiliations:** University of Maryland Baltimore, Baltimore, Maryland, United States; University of Maryland Baltimore, Baltimore, Maryland, United States

## Abstract

Even though the period following hospitalization is a critical time for physical activity, recently hospitalized older adults with dementia spend 83.3% of their days sedentary. Filling a research gap, this study aims to test the hypothesis that greater amounts of physical activity at one month post discharge is associated with lower mortality over the first year post discharge. We obtained the variable of “physical activity one month post discharge” through application of the MotionWatch8 accelerometer for 24 hours between 30-60 days after hospital discharge. We obtained the variable of “mortality status over one year post discharge” through care partners’ report of participant death or alive status at 6 months and 1 year post hospital discharge. We performed simple logistic regression and multiple logistic regression, controlling for comorbidities, age, and cognitive status. Among 42 recently discharged older adults with dementia, we found that participants’ amount of time spent in physical activity one month post discharge was not statistically significantly associated with mortality within one year post discharge (Simple Logistic Regression: OR=.996, CI=.992,1.000; p=.053; Multiple Logistic Regression: OR=.996, CI=.992,1.001; p=.095). However, we did observe a strong trend. In particular, the data suggests that as a recently discharged person with dementia increases their time spent in physical activity by 10 daily minutes, the odds that they will die over the next year decreases by 4%. This demonstrates the critical importance of physical activity following hospital discharge among older adults with dementia and the need to test this association in a larger sample.

